# Supplementary material for: Glucagon-like peptide-1 receptor agonist in large vessel occlusion treated by reperfusion therapy—a phase 2 randomized trial
Source: Nat Commun. 2025 Dec 14;16:11274. doi: 10.1038/s41467-025-66167-z (PMC12717234; doi:10.1038/s41467-025-66167-z)
Supplement: Supplementary file 1 — Supplementary Information [file 41467_2025_66167_MOESM1_ESM.pdf]

Table S1. Inclusion and exclusion criteria

**Inclusion criteria**
LVO stroke at terminal ICA or proximal M1 eligible for emergency endovascular treatment as per current treatment guideline
LKW-to-puncture time ≤ 12 hours
Age 18 years or greater
National Institute of Health Stroke Scale ≥ 10
LVO stroke due to thromboembolism or intracranial stenosis (acute or acute on chronic occlusion)
Patients who received computer tomographic angiography and perfusion
Pre-stroke (24 hours prior to stroke onset) independent functional status with modified Rankin Scale ≤ 2
Consent process completed as per national laws and regulation and the applicable ethics committee requirements
**Exclusion criteria**
ASPECT score ≤ 5
Intracranial hemorrhage on pre-EVT imaging
EVT completed before randomization
LVO etiologies other than thromboembolism or intracranial stenosis (acute or acute on chronic total occlusion), e.g. arterial dissection, infective endocarditis on initial diagnostic imaging.
Estimated or known body mass index < 18 kg/m^2
Pregnancy/Lactation; female, with positive urine or serum beta human chorionic gonadotropin (β-hCG) test, or breastfeeding.
Creatinine clearance < 30mL/min
Severe or fatal comorbid illness, e.g. terminal malignancy
Participation in another clinical trial investigating a drug, medical device, or a medical procedure in the 30 days preceding trial inclusion.
History of allergy to GLP-1RA
Family or personal history of multiple endocrine neoplasia, medullary thyroid carcinoma, pancreatic carcinoma, known proliferative diabetic retinopathy
Active sepsis on randomization
Patients with hypoglycaemia on presentation. Defined as capillary or serum glucose level of <4mmol/L.
Patient already on GLP-1RA prior to screening.
Contraindications to iodine-based CT contrast.

Abbreviation: LVO: large vessel occlusion; ICA: internal carotid artery; LKW: last-known-well; ASPECT: Alberta Stroke Program Early Computer Tomography Score; GLP-1RA: glucagon-like peptide-1 receptor agonist; CT: computer tomography.

Table S2. Characteristics of participants who were lost to follow-up vs. completed follow-up

	**Lost-to-follow-up (n=5)**	**Completed follow-up n=135)**
Age median(IQR)	51 (51, 74)	70 (61, 76)
Male sex n(%)	4 (80)	90 (66.7)
Active smoker n(%)	3 (60)	30 (22.2)
Body weight (kg) median(IQR)	78.5 (70.2, 82.5)	67.5 (60, 80)
Hypertension n(%)	5 (100)	85 (63)
Diabetes mellitus n(%)	2 (40)	25 (18.5)
Atrial fibrillation n(%)	1 (20)	36 (26.7)
Ischemic heart disease n(%)	1 (20)	15 (11.1)
Congestive heart failure n(%)	0 (0)	6 (4.4)
History of stroke n(%)	1 (20)	22 (16.3)
Peripheral vascular disease n(%)	0 (0)	2 (1.5)
Chronic kidney disease n(%)	0 (0)	2 (1.5)
Secondary diversion n(%)	1 (20)	14 (10.4)
Systolic blood pressure median(IQR)	156 (154, 168)	142 (128, 159.5)
Diastolic blood pressure median(IQR)	94 (85, 100)	84 (76, 90)
Blood glucose on admission (mmol/L) median(IQR)	6.3 (5.6, 10)	6.6 (5.6, 8.2)
HbA1c (%) median(IQR)	6.2 (5.9, 6.8)	5.9 (5.5, 6.3)
ASPECTS median(IQR)	8.5 (7.5, 9.2)	8 (7, 10)
Infarct core (mL) median(IQR)	15.5 (4.2, 40)	11.7 (3.1, 26.2)
Premorbid mRS median(IQR)	0 (0, 0)	0 (0, 0)
Baseline NIHSS (IQR)	13 (13, 19)	16 (12, 20)
LKW-to-puncture (mins) median(IQR)	505 (384, 810)	330 (193, 436.5)
General anesthesia n(%)	4 (80)	89 (65.9)
Intravenour thrombolysis n(%)	4 (80)	68 (50.4)
Collateral score median(IQR)	2 (1, 2)	2 (1, 2)
mTICI 2c or above (%)	4 (80)	109 (80.7)
Aspiration n(%)	3 (60)	61 (45.2)
Stent retriever n(%)	1 (20)	13 (9.6)
Combined aspiration/stent retriever n(%)	1 (20)	61 (45.2)
Acute intracranial stenting n(%)	1 (20)	40 (29.6)

Abbreviation: HbA1c: glycated hemoglobin A1c; ASPECTS: Alberta Stroke Program Early Computer Tomography Score; LKW: last-known-well; mTICI: modified Thrombolysis in Cerebral Infarction score; mRS: modified Rankin scale; NIHSS: National Institute of Health Stroke Scale.

Table S3. Details of Protocol Deviation

Subject no.	Randomization arm	Details of protocol deviation
004	Standard therapy	NIHSS 9 on presentation
007	Semaglutide	Onset-to-puncture time 18 hours
013	Semaglutide	Premorbid mRS 3

Abbreviation: NIHSS: National Institute of Health Stroke Scale, mRS: modified Rankin scale

Table S4. Interim Safety Analysis

Parameters	Semaglutide (n=43)	Standard Therapy (n=47)
Intracranial hemorrhage n(%)	2 (4.7)	11 (23.4)
Malignant brain edema n(%)	2 (4.7)	6 (12.8)
Change between baseline and D3 NIHSS median(IQR)	-8 (-12, -3.5)	-5 (-9.5, 1)
Death at 90 days n(%)*	4 (12.9)	8 (21)
mRS 4-6 at 90 days) n(%)*	10 (33.3)	15 (39.5)

Abbreviation: NIHSS: National Institutes of Health Stroke Scale; mRS: modified Rankin Scale.

** 31 patients in the semaglutide group and 38 patients in the standard therapy group completed 90-day mRS assessment at the time of interim analysis, 1 patient in the semaglutide was lost to follow-up and was imputed as death.*

Table S5A. Baseline characteristics of the no-IVT stratum

Parameters	Semaglutide (n=34)	Standard Therapy (n=34)
Age mean±sd	66.6 ± 11.8	65 ± 10.5
Male sex n(%)	22 (64.7)	22 (64.7)
Active smoker n(%)	5 (14.7)	6 (17.6)
Body weight (kg) mean±sd	66.6 ± 13.6	69 ± 12.5
Hypertension n(%)	21 (61.8)	23 (67.6)
Diabetes mellitus n(%)	5 (14.7)	6 (17.6)
Atrial fibrillation n(%)	14 (41.2)	5 (14.7)
Ischemic heart disease n(%)	5 (14.7)	3 (8.8)
Congestive heart failure n(%)	3 (8.8)	0 (0)
History of stroke n(%)	9 (26.5)	6 (17.6)
Peripheral vascular disease n(%)	1 (2.9)	0 (0)
Chronic kidney disease n(%)	0 (0)	0 (0)
Secondary diversion n(%)	0 (0)	0 (0)
Systolic blood pressure mean±sd	140.8 ± 15.9	148.8 ± 21.3
Diastolic blood pressure mean±sd	80.4 ± 12.3	85.8 ± 13.9
Blood glucose on admission (mmol/L) mean±sd	6.7 ± 1.6	7.6 ± 3.1
HbA1c (%) median(IQR)	5.7 (5.4, 6)	5.8 (5.5, 6.1)
ASPECTS median(IQR)	8 (7, 10)	8 (6.5, 9)
Infarct core (mL) median(IQR)	8 (1.5, 20.9)	14.7 (0.8, 23.2)
Premorbid mRS median(IQR)	0 (0, 0)	0 (0, 0)
Baseline NIHSS (IQR)	16 (11, 20.5)	16 (10.2, 18.8)
Collateral score median(IQR)	1 (1, 2)	2 (1, 2)
LKW-to-puncture (mins) median(IQR)	367.2 (256.8, 519)	354 (242.5, 501)
General anesthesia	18 (52.9)	26 (76.5)
mTICI 2c or above (%)	29 (85.3)	29 (85.3)
Aspiration n(%)	17 (50)	12 (35.3)
Stent retriever n(%)	2 (5.9)	3 (8.8)
Combined aspiration/stent retriever n(%)	15 (44.1)	19 (55.9)
Acute intracranial stenting n(%)	8 (23.5)	10 (29.4)

Table S5B. Secondary continuous outcomes of the no-IVT stratum

Secondary outcomes	Semaglutide (n=34)	Standard Therapy (n=34)
Change between baseline and D3 NIHSS median(IQR)	-8 (-12, -4)	-3.5 (-6, 0.8)
Final infarct size (mL) median(IQR)	11.1 (5.1, 36)	17.1 (9.8, 25.9)
Blood glucose on day 3 (mmol/L) mean±sd	6.4 ± 1.4	7.8 ± 3.1
Change between baseline and D3 glucose (mmol/L) mean±sd	-0.4 ± 1.2	0.2 ± 2.7

Abbreviation: HbA1c: glycated hemoglobin A1c; ASPECTS: Alberta Stroke Program Early Computer Tomography Score; LKW: last-known-well; mTICI: modified Thrombolysis in Cerebral Infarction score; mRS: modified Rankin scale; NIHSS: National Institute of Health Stroke Scale.

Table S6A. Baseline characteristics of the IVT stratum

Parameters	Semaglutide (n=35)	Standard Therapy(n=37)
Age mean±sd	71.9 ± 9	69.2 ± 11.4
Male sex n(%)	22 (62.9)	28 (75.7)
Active smoker n(%)	12 (34.3)	10 (27)
Body weight (kg) mean±sd	73.5 ± 15.7	67.6 ± 12.6
Hypertension n(%)	21 (60)	25 (67.6)
Diabetes mellitus n(%)	5 (14.3)	11 (29.7)
Atrial fibrillation n(%)	7 (20)	11 (29.7)
Ischemic heart disease n(%)	6 (17.1)	2 (5.4)
Congestive heart failure n(%)	2 (5.7)	1 (2.7)
History of stroke n(%)	6 (17.1)	2 (5.4)
Peripheral vascular disease n(%)	0 (0)	1 (2.7)
Chronic kidney disease n(%)	0 (0)	2 (5.4)
Secondary diversion n(%)	8 (22.9)	7 (18.9)
Systolic blood pressure mean±sd	152.6 ± 19.1	140.1 ± 25
Diastolic blood pressure mean±sd	86.4 ± 10.4	84.5 ± 14.6
Blood glucose on admission (mmol/L) mean±sd	7.4 ± 3.1	7.9 ± 3.3
HbA1c (%) median(IQR)	6 (5.6, 6.4)	6.1 (5.7, 6.6)
ASPECTS median(IQR)	8.5 (7, 9.2)	9 (7, 9.5)
Infarct core (mL) median(IQR)	13.9 (4.4, 44.9)	12 (4.5, 26.2)
Premorbid mRS median(IQR)	0 (0, 0)	0 (0, 0)
Baseline NIHSS (IQR)	16 (14, 19.5)	15 (13, 21)
Collateral score median(IQR)	1 (1, 2)	2 (1, 2)
LKW-to-puncture (mins) median(IQR)	263 (178, 419.5)	262 (155, 384)
General anethesia n(%)	23 (65.7)	26 (70.3)
mTICI 2c or above (%)	25 (71.4)	30 (81.1)
Aspiration n(%)	15 (42.9)	20 (54.1)
Stent retriever n(%)	6 (17.1)	3 (8.1)
Combined aspiration/stent retriever n(%)	14 (40)	14 (37.8)
Acute intracranial stenting n(%)	13 (37.1)	10 (27)

Table S6B. Secondary continuous outcomes of the IVT stratum

Secondary outcomes	Semaglutide (n=35)	Standard Therapy(n=37)
Change between baseline and D3 NIHSS median(IQR)	-8 (-12, 0)	-6 (-11, -3)
Final infarct size (mL) median(IQR)	25.9 (3.3, 122.5)	28.6 (11.3, 57.2)
Blood glucose on day 3 (mmol/L) mean±sd	7.7 ± 3.2	8 ± 3.2
Change between baseline and D3 glucose (mmol/L) mean±sd	0.7 ± 1.8	-0.1 ± 3.3

Abbreviations: HbA1c: glycated hemoglobin A1c; ASPECTS: Alberta Stroke Program Early Computer Tomography Score; LKW: last-known-well; mTICI: modified Thrombolysis in Cerebral Infarction score; mRS: modified Rankin scale; NIHSS: National Institute of Health Stroke Scale.

Table S7. Unadjusted Risk Ratios

	**Unadjusted RR (95% CI)**
Primary efficacy outcome (mRS 0–2 at 90 days)	1.02 (0.92–1.13)
Primary safety outcome	0.99 (0.89–1.11)
mRS 0–3 at 90 days	0.99 (0.90–1.09)
mRS 0–1 at 90 days	1.09 (0.97–1.22)
Death	1 (0.9–1.11)
Intracranial hemorrhage	0.92 (0.83–1.00)
Malignant brain edema	0.98 (0.90–1.06)

Abbreviation: mRS: modified Rankin scale, RR: risk ratio

N.B. Primary safety outcome was defined as a composite of death at 90 days, intracranial hemorrhage or malignant brain edema. Intracranial hemorrhage is defined as Heidelberg bleeding classification class 2 or above.

Table S8. Complete case analysis (overall study sample)

Parameters	Semaglutide (n=66)	Standard Therapy (n=69)	RR (95% CI)
Primary efficacy outcome (mRS 0–2 at 90 days) n(%)	39 (59.1)	39 (56.5)	1.05 (0.96–1.16)
Primary safety outcome n(%)	13 (19.7)	15 (21.7)	0.98 (0.88–1.09)
Intracranial hemorrhage n(%)	4 (6.1)	10 (14.5)	0.92 (0.84–1.01)
Malignant brain edema n(%)	5 (7.6)	7 (10.1)	0.97 (0.89–1.06)
Death n(%)	8 (12.1)	9 (13)	0.99 (0.89–1.09)
mRS 0–1 at 90 days n(%)	27 (40.9)	21 (30.4)	1.2 (1.03–1.39)
mRS 0–3 at 90 days n(%)	44 (66.7)	67 (68.1)	1.01 (0.92–1.11)
Change between baseline and D3 NIHSS median(IQR)	-8 (-12, -2)	-4 (-9, -1)	
Final infarct size (mL) median(IQR)	16.9 (5.4, 81.1)	21.8 (10.8, 39.9)	
Blood glucose on day 3 (mmol/L) mean±sd	7 ± 2.6	7.8 ± 3	
Change between baseline and D3 glucose (mmol/L) mean±sd	0.1 ± 1.6	0.1 ± 3.1	

Abbreviation: RR: risk ratio, mRS: modified Rankin scale, NIHSS: National Institute of Health Stroke Scale

N.B. Primary safety outcome was defined as a composite of death at 90 days, intracranial hemorrhage or malignant brain edema. Intracranial hemorrhage is defined as Heidelberg bleeding classification class 2 or above.

Table S9. Complete case analysis (no-IVT stratum)

Parameter	Semaglutide (n=33)	Standard Therapy (n=34)	RR (95% CI)
Primary efficacy outcome (mRS 0–2 at 90 days) n(%)	22 (66.7)	15 (44.1)	1.19 (1.04–1.37)
Primary safety outcome n(%)	6 (18.2)	10 (29.4)	0.89 (0.76–1.04)
Intracranial hemorrhage n(%)	3 (9.1)	6 (17.6)	0.91 (0.79–1.05)
Malignant brain edema n(%)	2 (6.1)	5 (14.7)	0.91 (0.8–1.03)
Death n(%)	2 (6.1)	8 (23.5)	0.84 (0.73–0.97)
mRS 0–1 at 90 days n(%)	15 (45.5)	9 (26.5)	1.21 (1.05–1.39)
mRS 0–3 at 90 days n(%)	25 (75.8)	19 (55.9)	1.16 (1.01–1.32)
Change between baseline and D3 NIHSS median(IQR)	-8 (-12, -4)	-3.5 (-6, 0.8)	
Final infarct size (mL) median(IQR)	13.1 (6.2, 38.7)	17.1 (9.8, 25.9)	
Blood glucose on day 3 (mmol/L) mean±sd	6.4 ± 1.4	7.8 ± 3.1	
Change between baseline and D3 glucose (mmol/L) mean±sd	-0.5 ± 1.1	0.2 ± 2.7	

Abbreviation: RR: risk ratio, mRS: modified Rankin scale, NIHSS: National Institute of Health Stroke Scale

N.B. Primary safety outcome was defined as a composite of death at 90 days, intracranial hemorrhage or malignant brain edema. Intracranial hemorrhage is defined as Heidelberg bleeding classification class 2 or above.

Table S10. **Complete case analysis (IVT stratum)**

Parameters	Semaglutide (n=33)	Standard Therapy (n=35)	RR (95% CI)
Primary efficacy outcome (mRS 0–2 at 90 days) n(%)	17 (51.5)	24 (68.6)	0.95 (0.84–1.06)
Primary safety outcome n(%)	7 (21.2)	5 (14.3)	1.07 (0.94–1.23)
Intracranial hemorrhage n(%)	1 (3)	4 (11.4)	0.93 (0.83–1.03)
Malignant brain edema n(%)	3 (9.1)	2 (5.7)	1.03 (0.92–1.16)
Death n(%)	6 (18.2)	1 (2.9)	1.15 (0.98–1.3)
mRS 0–1 at 90 days n(%)	12 (36.4)	12 (34.3)	1.05 (0.9–1.22)
mRS 0–3 at 90 days n(%)	19 (57.6)	28 (80)	0.89 (0.79–1.01)
Change between baseline and D3 NIHSS median(IQR)	-8 (-12, 0)	-5 (-11, -2.5)	
Final infarct size (mL) median(IQR)	27.7 (5.3, 127.6)	28.6 (12.4, 59.1)	
Blood glucose on day 3 (mmol/L) mean±sd	7.6 ± 3.3	7.8 ± 2.9	
Change between baseline and D3 glucose (mmol/L) mean±sd	0.7 ± 1.8	-0.1 ± 3.4	

Abbreviation: RR: risk ratio**,** mRS: modified Rankin scale, NIHSS: National Institute of Health Stroke Scale

N.B. Primary safety outcome was defined as a composite of death at 90 days, intracranial hemorrhage or malignant brain edema. Intracranial hemorrhage is defined as Heidelberg bleeding classification class 2 or above.

Figure S1. Association between last-known-well (LKW) to puncture time and risk ratios of the achieving modified Rankin Scale (mRS) 0–2 at 90 days

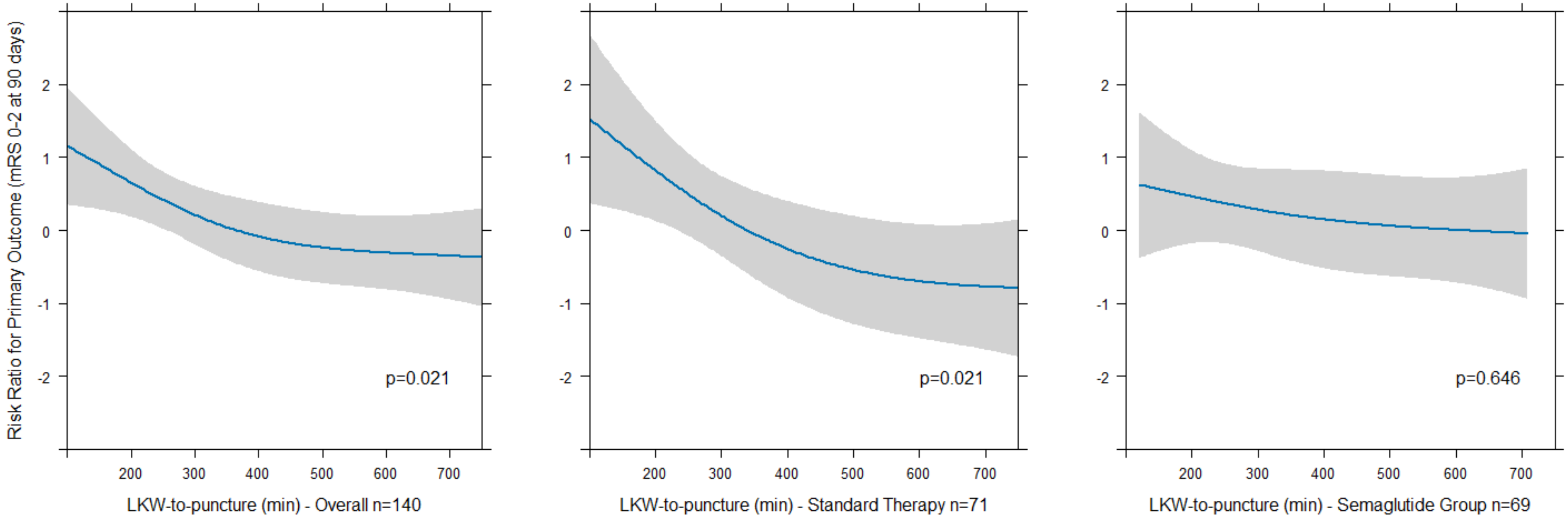

Supplementary Note 1: Summary of Protocol and Statistical Analysis Plan Changes

The following is a list of main protocol changes from protocol version 1.0 dated 16 June 2023 to version 2.0 dated 15 May 2024. The main reasons for the protocol changes are as follows:

**Section**	**Protocol version 1.0 change from**	**Protocol version 2.0 change to**	**Rationale**
Methods (Outcomes)	The primary outcome is good functional outcome, defined as modified Rankin Scale 0-3 at 90 days.	The primary outcome is good functional outcome, defined as modified Rankin Scale 0-2 at 90 days. Secondary outcomes include modified Rankin Scale 0-1, 0-3, and ordinal shift at 90 days.	Most clinical trials adopted mRS 0 to 2 as the definition for good neurological recovery for anterior circulation large vessel occlusion (1-3).
Methods (Statistical Analysis)	Multivariable logistic regression for binary outcomes.	Multivariable modified Poisson regression for binary outcomes.	Compared to logistic regression, modified Poisson regression could provide unbiased estimation of risk ratios in the setting of common event occurrence (4).

Supplementary reference

1. Saver JL, Goyal M, Bonafe A, Diener HC, Levy EI, Pereira VM, et al. Stent-retriever thrombectomy after intravenous t-PA vs. t-PA alone in stroke. N Engl J Med. 2015;372(24):2285-95.
2. Berkhemer OA, Fransen PS, Beumer D, van den Berg LA, Lingsma HF, Yoo AJ, et al. A randomized trial of intraarterial treatment for acute ischemic stroke. N Engl J Med. 2015;372(1):11-20.
3. Goyal M, Demchuk AM, Menon BK, Eesa M, Rempel JL, Thornton J, et al. Randomized assessment of rapid endovascular treatment of ischemic stroke. N Engl J Med. 2015;372(11):1019-30.
4. Chen W, Qian L, Shi J, Franklin M. Comparing performance between log-binomial and robust Poisson regression models for estimating risk ratios under model misspecification. BMC Med Res Methodol. 2018;18(1):63.
