# Supplementary material for: Glucagon-like peptide-1 receptor agonist in large vessel occlusion treated by reperfusion therapy—a phase 2 randomized trial
Source: Nat Commun. 2025 Dec 14;16:11274. doi: 10.1038/s41467-025-66167-z (PMC12717234; doi:10.1038/s41467-025-66167-z)
Supplement: Supplementary file 2 — Reporting Summary [file 41467_2025_66167_MOESM2_ESM.pdf]

nature portfolio

Corresponding author(s): Bonaventure Y. Ip

Last updated by author(s): Mar 8, 2025

Reporting Summary

Nature Portfolio wishes to improve the reproducibility of the work that we publish. This form provides structure for consistency and transparency in reporting. For further information on Nature Portfolio policies, see our Editorial Policies and the Editorial Policy Checklist.

Statistics

For all statistical analyses, confirm that the following items are present in the figure legend, table legend, main text, or Methods section.

n/a	Confirmed	
☐	☒	The exact sample size (*n*) for each experimental group/condition, given as a discrete number and unit of measurement
☐	☒	A statement on whether measurements were taken from distinct samples or whether the same sample was measured repeatedly
☐	☒	The statistical test(s) used AND whether they are one- or two-sided<br>*Only common tests should be described solely by name; describe more complex techniques in the Methods section.*
☐	☒	A description of all covariates tested
☐	☒	A description of any assumptions or corrections, such as tests of normality and adjustment for multiple comparisons
☐	☒	A full description of the statistical parameters including central tendency (e.g. means) or other basic estimates (e.g. regression coefficient) AND variation (e.g. standard deviation) or associated estimates of uncertainty (e.g. confidence intervals)
☐	☒	For null hypothesis testing, the test statistic (e.g. *F*, *t*, *r*) with confidence intervals, effect sizes, degrees of freedom and *P* value noted<br>*Give P values as exact values whenever suitable.*
☒	☐	For Bayesian analysis, information on the choice of priors and Markov chain Monte Carlo settings
☒	☐	For hierarchical and complex designs, identification of the appropriate level for tests and full reporting of outcomes
☒	☐	Estimates of effect sizes (e.g. Cohen's *d*, Pearson's *r*), indicating how they were calculated

Our web collection on statistics for biologists contains articles on many of the points above.

Software and code

Policy information about availability of computer code

Data collection	Microsoft excel
Data analysis	R studio (v4.4.1, R Project for Statistical Computing, RStudio Team 2022), blockrand package v1.5 for randomization

For manuscripts utilizing custom algorithms or software that are central to the research but not yet described in published literature, software must be made available to editors and reviewers. We strongly encourage code deposition in a community repository (e.g. GitHub). See the Nature Portfolio guidelines for submitting code & software for further information.

Data

Policy information about availability of data

All manuscripts must include a data availability statement. This statement should provide the following information, where applicable:

- Accession codes, unique identifiers, or web links for publicly available datasets
- A description of any restrictions on data availability
- For clinical datasets or third party data, please ensure that the statement adheres to our policy

The data generated in the figures have been deposited in the Figshare database (https://doi.org/10.6084/m9.figshare.28089197). Anonymized data, including age, sex, admission NIHSS, premorbid mRS, treatment allocation, and study outcomes will be made available by requesting the corresponding authors (Bonaventure Y. Ip, email: ipyiuming@gmail.com, or Ho Ko, email: ho.ko@cuhk.edu.hk) from qualified investigators for academic purposes, beginning 3 months and ending 5 years following publication. The corresponding authors will reply to the request within 2 months, subject to the approval of the Joint Chinese University of Hong Kong-New Territory East Cluster and the Linyi People’s Hospital Clinical Research Ethics Committees. Source data are provided with this paper.

Research involving human participants, their data, or biological material

Policy information about studies with human participants or human data. See also policy information about sex, gender (identity/presentation), and sexual orientation and race, ethnicity and racism.

Reporting on sex and gender	The number and percentage of sex were reported.
Reporting on race, ethnicity, or other socially relevant groupings	All study participants were ethnic Chinese.
Population characteristics	The mean age of patients was 68.2 ± 10.9 years, with 94 (67.1%) male and 46 (32.9%) female subjects. The median (interquartile range) National Institutes of Health Stroke Scale (NIHSS) was 16 (12, 20) and the baseline Alberta Stroke Program Early Computed Tomography Score (ASPECTS) was 8 (7, 10). The median time (interquartile range) for onset to puncture was 333 (194, 440) minutes. Intravenous thrombolysis (IVT), either by alteplase 0.9 mg/kg or tenecteplase 0.25 mg/kg, was given to 35 (50.7%) in the semaglutide group and 37 (52.1%) in the standard therapy group.
Recruitment	Consecutive patients with large vessel occlusion stroke undergoing endovascular therapy who fulfilled the inclusion and exclusion criteria, and consented to the study.<br>Potential bias:<br>1. Since the study recruited patients with large vessel occlusions with a National Institutes of Health Stroke Scale or 10 or above, the study may not be applicable to patients with milder strokes.<br>2. All study participants are Chinese, the study findings may not be generalized to other ethnicities.
Ethics oversight	Joint Chinese University of Hong Kong-New Territories East Cluster Clinical Research Ethics Committee; Science Research Ethics Committee, Linyi People's Hospital

Note that full information on the approval of the study protocol must also be provided in the manuscript.

Field-specific reporting

Please select the one below that is the best fit for your research. If you are not sure, read the appropriate sections before making your selection.

☒ Life sciences ☐ Behavioural & social sciences ☐ Ecological, evolutionary & environmental sciences

For a reference copy of the document with all sections, see nature.com/documents/nr-reporting-summary-flat.pdf

Life sciences study design

All studies must disclose on these points even when the disclosure is negative.

Sample size	No human trials had evaluated the safety and efficacy of GLP-1RA on EVT-eligible patients. We hypothesized that semaglutide had a mild-to-moderate effect size, corresponding to a standardized difference of 0.15-0.25, in reducing poor functional outcome in patients eligible for the study. When treating the primary outcome (mRS 0-2 at 90 days) as a binary outcome variable, a sample size of 120 for the phase 2 trial is recommended in order to guard against the lack of precision by using inflated estimates. Considering a rate of 2.5% suboptimal scan qualities, 5% of loss-to-follow-up and 10% of suboptimal recanalization according to the track records of participating centers, 140 participants were required for 90% power and significance level of 0.05 for the main trial. Details of the sample size estimation are described in the Study Protocol.
Data exclusions	No data were excluded in the primary intention-to-treat analyses. Complete case analyses for the outcomes were performed in patients without missing data (n=5).
Replication	The Methods section and the Study Protocol provide sufficient details to replicate the study.
Randomization	Patients were then randomized in a 1:1 ratio to receive semaglutide plus endovascular therapy or endovascular therapy alone in the emergency department. Permuted blocked randomization was employed in the study to ensure the balance of subjects throughout the trial setting. Randomly generated block sizes of 2, 4, and 6 were adopted to avoid possible mid-block inequality caused by larger blocks. Two blocks with unbalanced treatment distribution were generated at the start and middle of the list. The randomization process was performed using the blockrand package (v1.5) in R studio (v4.4.1, R Project for Statistical Computing, RStudio Team 2022). Treatment allocation was concealed until study eligibility was confirmed by two investigators and informed consent had been obtained.
Blinding	Treatment allocation were blinded from chief interventionalists, clinical and radiological outcome assessors as per a prospective, randomized, open-label, blinded endpoint trial.

Reporting for specific materials, systems and methods

We require information from authors about some types of materials, experimental systems and methods used in many studies. Here, indicate whether each material, system or method listed is relevant to your study. If you are not sure if a list item applies to your research, read the appropriate section before selecting a response.

Materials & experimental systems

n/a	Involved in the study	
☒	☐	Antibodies
☒	☐	Eukaryotic cell lines
☒	☐	Palaeontology and archaeology
☒	☐	Animals and other organisms
☐	☒	Clinical data
☒	☐	Dual use research of concern
☒	☐	Plants

Methods

n/a	Involved in the study	
☒	☐	ChIP-seq
☒	☐	Flow cytometry
☒	☐	MRI-based neuroimaging

Clinical data

Policy information about clinical studies

All manuscripts should comply with the ICMJE guidelines for publication of clinical research and a completed CONSORT checklist must be included with all submissions.

Clinical trial registration

NCT05920889

Study protocol

The study protocol has been uploaded as a supplementary material.

Data collection

Data pre-specified in the Study Protocol was collected prospectively from the recruitment of the first study participant (August 11 2023) to study end date (25 October 2024), when the 90 day modified Rankin Scale was obtained for the last study participant. All radiological and clinical outcome assessors were blinded from treatment allocation.

Data collection was conducted in the stroke wards or neurointensive care unit during a participant's in-patient stay. Clinical follow-up was arranged 90 days after randomization for evaluation of functional recovery. A telephone or video conferencing follow-up was arranged for patients who were unable to attend an in-person follow-up.

Outcomes

The primary efficacy outcome was good neurological recovery, defined as modified Rankin Scale (mRS) of 0 to 2 at 90 days. The primary safety outcome was a composite of death, malignant brain edema (MBE), any intracranial hemorrhage (ICH). Secondary outcomes were the ordinal shift of mRS at 90 days, mRS 0 to 3 at 90 days, mRS 0 to 1 at 90 days, death, final infarct size, MBE, and ICH. Secondary exploratory outcomes included changes between day-3 and baseline National Institute of Health Stroke Scale (NIHSS) and day-3 and baseline blood glucose level.

Definition of endpoints:

1. mRS is a 0 to 6 point ordinal scale for measuring disability after stroke. Functional dependence increases with each point of increment, with 6 indicating death.
2. MBE is defined as parenchymal hypodensity of at least 50% of the middle cerebral artery territory and signs of local brain swelling such as sulcal effacement and compression of the lateral ventricle, and midline shift of ≥5 mm at the septum pellucidum or pineal gland with obliteration of the basal cisterns.
3. ICH is defined as Heidelberg bleeding classification class 2 or above after the procedure.
4. Infarct size was quantified from brain magnetic resonance imaging.
5. NIHSS is a 0 to 42 point scale for measuring severity of stroke. Severity increases with each point of increment.

Plants

Seed stocks

Not applicable

Novel plant genotypes

Not applicable

Authentication

Not applicable
